# Recrudescence of Natalizumab‐Induced Pneumonitis and Peripheral Hypereosinophilia: Case Report and Literature Review

**DOI:** 10.1002/rcr2.70191

**Published:** 2025-04-25

**Authors:** Gerardo Arwi, Louis Chhor, Vivek Malipatil

**Affiliations:** ^1^ Department of Respiratory and Sleep Medicine Eastern Health Melbourne Victoria Australia

**Keywords:** hypereosinophilia, natalizumab, pneumonitis

## Abstract

We report a rare case of recrudescence of natalizumab‐induced pneumonitis and peripheral hypereosinophilia, hence uniquely satisfying the re‐exposure principle outlined by the Naranjo Adverse Drug Reaction Probability Scale. A 57‐year‐old female with relapsing–remitting multiple sclerosis presented with acute respiratory symptoms, eosinophilia of 2.08 × 10^9^/L, and chest computed tomography showing consolidation of the right upper and lower lobes. Her septic and autoimmune work‐up was negative. She did not improve with broad‐spectrum antibiotics. Bronchoalveolar lavage revealed 75% lymphocytes. Upon discussion at a multi‐disciplinary meeting, her antibiotics were discontinued and she was commenced on prednisolone, which resulted in immediate improvement. She experienced two further recurrences of pneumonitis during her prednisolone tapering. After a two‐year remission, she was re‐started on natalizumab due to adverse events related to alternative biologic agents. Her pneumonitis and peripheral hypereosinophilia recurred. She responded well to another course of prednisolone, and subsequent follow‐up revealed complete recovery off natalizumab.

## Graphical abstract

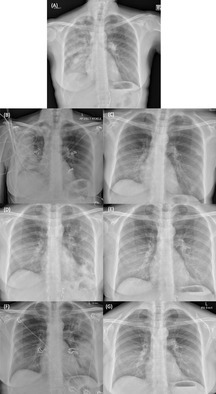

## 1 Introduction

Pneumonitis and peripheral hypereosinophilia are rare but documented complications of natalizumab. We report a rare case of recrudescence of natalizumab‐induced pneumonitis and peripheral hypereosinophilia, thereby uniquely satisfying the re‐exposure principle outlined by the Naranjo Adverse Drug Reaction Probability Scale. This strengthens the evidence of rare but serious side effects of natalizumab, emphasising the importance of monitoring for respiratory symptoms and peripheral eosinophilia in patients receiving natalizumab, particularly those with an underlying atopic predisposition or a history of drug‐induced pneumonitis and eosinophilia.

## 2 Case Report

**FIGURE 1:**
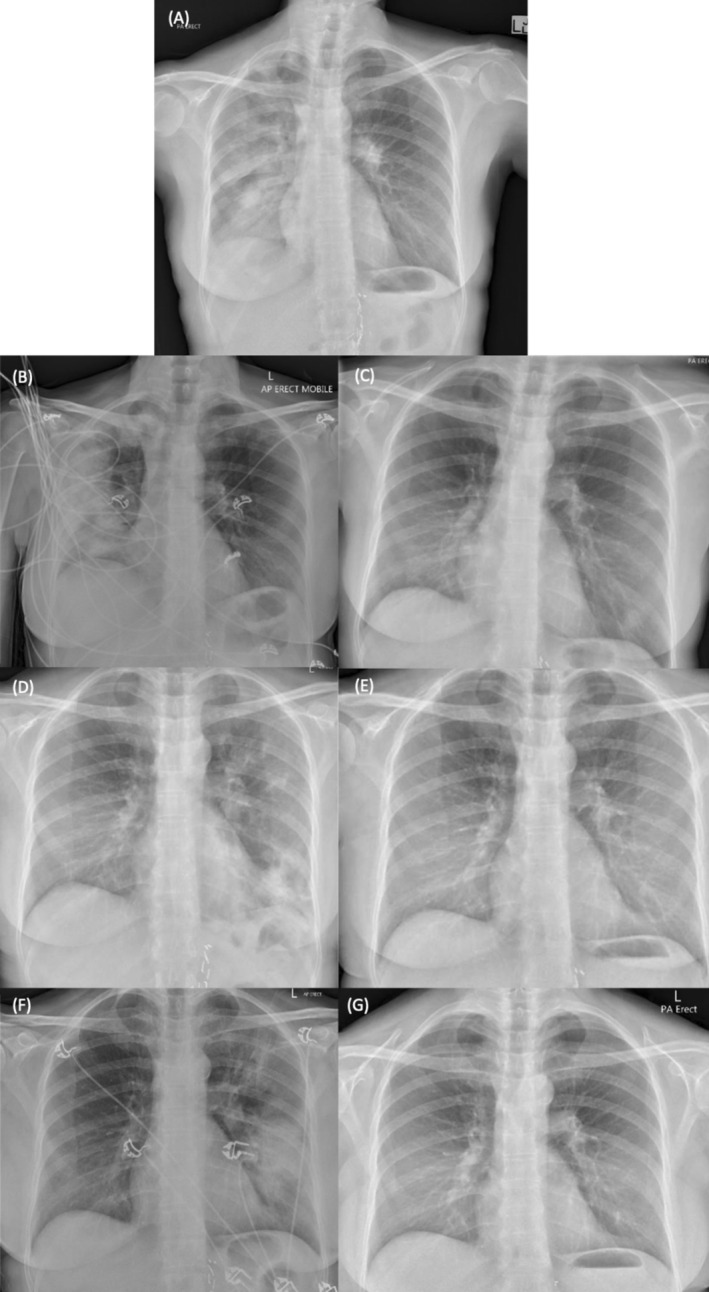
(A) Chest radiograph on initial admission demonstrating widespread consolidation throughout the right lung. (B) Radiograph obtained during the first recurrence revealing peripheral consolidation in the right lung. (C) Radiograph obtained after 3‐month prednisolone taper, showing resolution of the previous consolidation. (D) Radiograph obtained during the second recurrence showing irregular opacities in the left lower lobe. (E) Radiograph obtained after a 7‐month prednisolone taper, demonstrating resolution of the opacities. (F) Radiograph obtained during a recrudescence of disease 2 years later showing confluent opacities in the left mid‐zone and perihilar region of the left upper zone. (G) Radiograph obtained 4 months later during prednisolone tapering, showing resolution of opacities.

**FIGURE 2:**
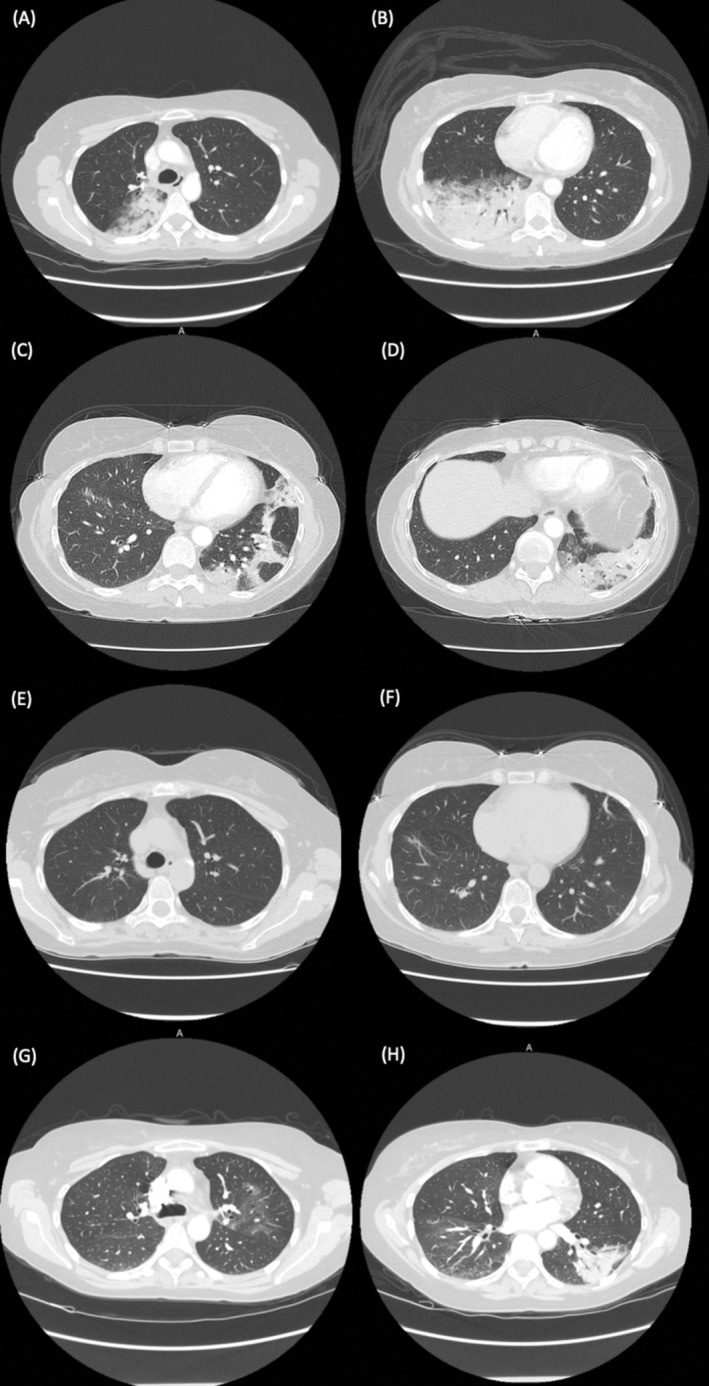
(A, B). Chest computed tomography (CT) scan axial cuts on initial admission, showing consolidation affecting the right upper and lower lobes. (C, D). Recurrence after 3‐month prednisolone taper demonstrated by consolidation in the left lower lobe and lingula, with associated ground‐glass opacities. (E, F). Complete resolution of consolidation following a 7‐month prednisolone taper. (G, H). Recrudescence of pneumonitis 2 years later, with consolidation in the left lower lobe and ground‐glass changes in the left upper lobe, lingula, and right lower lobe.

A 57‐year‐old female presented to the emergency department with worsening dry cough, shortness of breath, and fever over the past 4 days. Her past medical history includes relapsing–remitting multiple sclerosis (RRMS) currently being treated with monthly natalizumab infusions for the past 2 years without prior adverse reactions. She is a never‐smoker. On examination, her vital signs were normal with an oxygen saturation of 96% on room air, and physical examination revealed coarse crepitations in the right lower zone. There were no new neurological findings. Initial blood tests revealed a white cell count of 16.8 × 10^9^/L, eosinophilia of 2.08 × 10^9^/L, neutrophilia of 10.6 × 10^9^/L, and a C‐reactive protein of 303.5 mg/L. Chest radiographs and computed tomography (CT) scan of the chest showed consolidation of the right upper and lower lobes (Figures 1A and 2A,B). Broad‐spectrum antibiotics were initiated for community‐acquired bacterial pneumonia. However, she exhibited persistent respiratory symptoms and fever, raised inflammatory markers including eosinophilia, and consolidation on serial chest radiographs over the next 2 weeks.

A comprehensive septic workup including blood, urine, and stool cultures, as well as CT abdomen and pelvis, did not reveal additional sources of infection. Viral hepatitis, HIV, cytomegalovirus (CMV) and Epstein–Barr Virus (EBV) serology, and tuberculosis interferon gamma‐release assay were negative. Aspergillus precipitins, schistosomiasis and strongyloides serologies, as well as extended viral screen (including COVID and influenza) were negative. Autoimmune serology including anti‐nuclear antibodies, extractable nuclear antigen (ENA) antibodies and anti‐neutrophil cytoplasmic antibodies (ANCA) were unremarkable.

**FIGURE 3:**
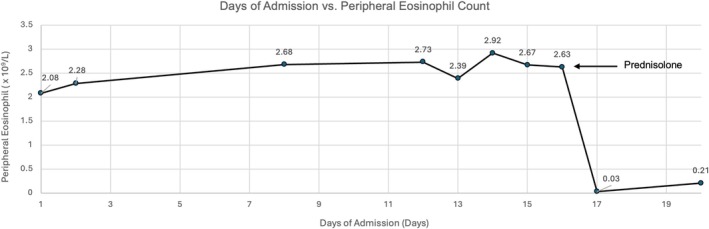
Peripheral eosinophil count throughout first admission.

Bronchoscopy was performed and a bronchoalveolar lavage (BAL) of the right lower lobe was obtained. Cell count differential revealed 75% lymphocytes and 5% eosinophils, and 12% neutrophils. No malignant cells were identified on cytology, and cultures including acid‐fast bacilli were negative. Differentials of drug‐induced pneumonitis, eosinophilic pneumonia, and organising pneumonia were suspected. Upon discussion at an interstitial lung disease (ILD) multi‐disciplinary meeting (MDM), her antibiotics were discontinued, and she was commenced on prednisolone 50 mg daily with a view to wean based on clinical response. This resulted in immediate clinical and biochemical improvement, with a reduction in eosinophilia from 2.6 × 10^9^/L to 0.03 × 10^9^/L following a single dose (Figure 3). She was discharged with a 6‐week tapering course of prednisolone and discontinuation of natalizumab.

Subsequent clinic reviews initially showed improvement in her clinical status, peripheral eosinophil count, and radiological findings. However, she experienced two further recurrences of pneumonitis over the following 6 months, each occurring shortly after completion of her tapering prednisolone course. The first recurrence occurred 3 weeks after the completion of her 6‐week prednisolone taper. She presented to the emergency department with exertional dyspnoea and lethargy, an eosinophil count of 0.26 × 10^9^/L, and a chest radiograph showing widespread right‐sided consolidation (Figure 1B). Prednisolone 50 mg daily was initiated, resulting in a good clinical response. She was subsequently discharged with a 3‐month tapering plan. Her infiltrates had improved on CXR (Figure 1C).

The second recurrence occurred 1 week after completing the 3‐month taper. She presented again with worsening exertional dyspnoea and was found to have mild eosinophilia of 0.58 × 10^9^/L. Chest radiograph shows irregular opacities in the left lower lobe and CT chest revealed consolidation in the left lower lobe and lingula, as well as ground‐glass opacities in the left upper and lower lobes (Figures 1D and 2C,D). Prednisolone 50 mg daily was reinitiated, followed by a 7‐month tapering regimen. She ultimately achieved remission, with chest radiograph and CT chest performed 6 months after the last recurrence showing complete resolution of the consolidative changes (Figures 1E and 2E,F). The diagnosis of natalizumab‐induced pneumonitis remained uncertain, given the absence of natalizumab as a trigger for these two episodes.

After 2 years of remission, she was reinitiated on natalizumab due to persistent symptoms of RRMS and intolerance of alternate MS agents. She remained in remission for a further 15 months before presenting again with shortness of breath, dry cough, and fever. Initial blood tests revealed eosinophilia of 0.79 × 10^9^/L. Initial chest radiograph shows confluent opacities in the left mid‐zone and perihilar region of the left upper zone (Figure 1F) and CT chest showed consolidation in the left lower lobe and ground‐glass changes in the left upper lobe, lingula, and right lower lobe (Figure 2G,H). Given her previous history, she was commenced on a course of prednisolone, which resolved both her symptoms and eosinophilia. There was suspicion that her respiratory symptoms were partly contributed by a flare of her MS causing diaphragmatic dysfunction; however, this was deemed unlikely as a respiratory function tests performed during the acute episode showed normal maximal inspiratory pressure of 63 cmH_2_O. She was discharged on a tapering course of prednisolone, and subsequent follow‐ups revealed complete recovery clinically and radiographically after permanent discontinuation of natalizumab (Figure 1G).

## 3 Discussion

Natalizumab is a monoclonal antibody that binds to the α4 subunit of α4β1 and α4β7 integrins on leukocytes, thereby preventing leukocyte trafficking into the central nervous system and reducing inflammation associated with conditions such as multiple sclerosis [1]. Recognised complications of natalizumab include hypersensitivity reactions, hepatotoxicity, thrombocytopenia, and progressive multifocal leukoencephalopathy [2].

Natalizumab‐induced pneumonitis and peripheral hypereosinophilia are rare but documented complications of this medication. There are only three other reported cases of these associations [3, 4, 5], as summarised in Table 1. Several hypotheses have been proposed to explain their pathogenesis. Binding of natalizumab to α4β1 and α4β7 integrins may induce endothelial injury, leading to the release of IL‐4 and IL‐13, which in turn promote the expression of vascular cell adhesion molecule (VCAM)‐1 and recruit eosinophils into the lung [6]. These can then trigger a T helper 2 (Th2) cells immune response that produces IL‐5, which subsequently primes the existing eosinophils and enhances their survival [5]. Other factors implicated in the pathological process include IL‐33, vascular endothelial growth factor (VEGF), leukotriene B4, and periostin [6].

**TABLE 1:** Summary of reported cases of natalizumab‐induced pneumonitis with peripheral hypereosinophilia.

	Our case	Case 1 [3]	Case 2 [4]	Case 3 [5]
Age	57 years	39 years	41 years	39 years
Sex	Female	Female	Female	Male
Country	Australia	Japan	Spain	UK
Relevant medical history	RRMS	RRMS Asthma	RRMS Allergic rhinoconjunctivitis *Dermatophagoides pteronyssinus* hypersensitivity	RRMS Crohn's disease
Smoking history	Never smoker	Never smoker	Smoker	Smoker
Natalizumab commencement	Commenced 2 years prior	Commenced 5 months prior	Commenced 1 year prior	Commenced 6 months prior
Prior MS treatment	None	Interferon beta (discontinued due to diagnoses of eosinophilic pneumonia needing steroid treatment) Fingolimod (changed to natalizumab due to persistent MS activity)	Interferon (changed to natalizumab due to persistent MS activity)	None
Peak eosinophil count	2.9 × 10^9^/L	5.2 × 10^9^/L	1.4 × 10^9^/L	2.87 × 10^9^/L
CT findings	Consolidation affecting the right upper and lower lobes	Bilateral patchy consolidation surrounded by ground‐glass opacity	Bilateral diffuse ground‐glass attenuation in association with intralobular lines and some bronchiectasis	Extensive bronchocentric and peripheral ground glass opacification with superimposed interlobular septal thickening
BAL eosinophil percentage	5%	35%	12%	Not reported
Outcome	Recovery with natalizumab withdrawal and prednisolone. Recrudescence of disease after re‐introduction of natalizumab. Subsequent recovery with natalizumab withdrawal and prednisolone.	Recovery with natalizumab withdrawal	Recovery with natalizumab withdrawal and inhaled corticosteroid (without systemic corticosteroid)	Recovery with natalizumab withdrawal and prednisolone

Extrapolating from the above hypotheses, patients with underlying asthma or allergies may be predisposed to natalizumab‐induced pneumonitis and peripheral hypereosinophilia, though our case did not have pre‐existing asthma. Due to the scarcity of documented cases, it is difficult to ascertain other definitive risk factors for this condition; however, certain characteristics, such as cigarette smoking, personal history of drug‐induced eosinophilia, and family history of eosinophilia, have been stipulated to increase susceptibility to these complications [7, 8].

Diagnosis of natalizumab‐induced pneumonitis and peripheral hypereosinophilia is based on a combination of clinical features, laboratory results, and radiological findings, with an appropriate temporal relationship with natalizumab exposure. Peripheral hypereosinophilia (above 2 × 10^9^/L), particularly within the first year of treatment, should raise suspicion for these complications [9]. Reported CT chest findings include patchy consolidations, broncho‐centric and peripheral ground‐glass opacifications, interlobular septal thickening, and bronchiectasis. Bronchoalveolar lavage cell‐count differentials may show variable levels of eosinophilia. Importantly, the diagnosis is made by excluding infections, other drug‐induced complications, and autoimmune disorders.

Mainstay treatment for natalizumab‐induced pneumonitis and peripheral hypereosinophilia involves discontinuation of natalizumab and initiation of corticosteroid therapy [10]. In some cases, inhaled corticosteroids and bronchodilators may also provide symptomatic relief. It is crucial to monitor the patient's clinical, biochemical, and radiological status to ensure resolution of pneumonitis and peripheral hypereosinophilia.

This is the first documented case of recrudescence of natalizumab‐induced pneumonitis and peripheral hypereosinophilia occurring in two separate instances in the same patient, thereby uniquely satisfying the re‐exposure principle outlined by Naranjo et al. in the Adverse Drug Reaction Probability Scale [11]. This further strengthens the evidence of a rare but serious side effect of natalizumab. Henceforth, monitoring for respiratory symptoms and peripheral eosinophilia is critical in patients receiving natalizumab, particularly those with an underlying atopic predisposition or history of drug‐induced pneumonitis and eosinophilia. As the use of natalizumab increases in clinical practice, post‐marketing surveillance and collection of similar case reports are essential to ensure the safety of this medication.

In conclusion, pneumonitis and peripheral hypereosinophilia are rare but documented complications of natalizumab. This report highlights the importance of monitoring for respiratory symptoms and peripheral eosinophilia in patients receiving natalizumab, particularly those with an underlying atopic predisposition or history of drug‐induced pneumonitis and eosinophilia.

## Author Contributions

Gerardo Arwi, Louis Chhor, and Vivek Malipatil contributed to the concept, clinical data collection, drafting, final review, and editing of the manuscript.

## Ethics Statement

The authors declare that appropriate written informed consent was obtained for the publication of this manuscript and accompanying images.

## Conflicts of Interest

The authors declare no conflicts of interest.

## Data Availability

The data that support the findings of this study are available on request from the corresponding author. The data are not publicly available due to privacy or ethical restrictions.
